# Integrative analysis of gut microbiota and plasma metabolites reveals mechanisms underlying aggressive behavior in chronically stressed broiler chickens

**DOI:** 10.1007/s44154-026-00297-2

**Published:** 2026-04-20

**Authors:** Xuanfu Wu, Jiaolong Zhang, Hongrui Ren, Xiaoxian Cheng, Jiang Gao, Wenqiang Ma

**Affiliations:** 1 Key Laboratory of Animal Physiology and Biochemistry, Ministry of Agriculture and Rural Affairs, College of Veterinary Medicine, Nanjing Agricultural University, Nanjing, Jiangsu 210095 China; 2 MOE Joint International Research Laboratory of Animal Health & Food Safety, Nanjing Agricultural University, Nanjing, Jiangsu 210095 China

**Keywords:** Chronic stress, Broiler chickens, Aggressive behavior, 16s rDNA, Metabolomics

## Abstract

Chronic stress in livestock production affects animal welfare and productivity, often leading to aggressive behavior in broiler chickens. We exposed broiler chickens to chronic corticosterone to simulate stress and compared them to controls, evaluating production performance, hypothalamic serotonin levels, cecal microbiota, plasma metabolites, and aggression. In this study, broiler chickens were divided into two groups following corticosterone injection: the control group (CON) and the chronic corticosterone exposure group (CORT). Stressed chickens showed reduced growth, lower hypothalamic serotonin, and increased aggression. Gut microbiota changes included increased *Clostridium* and decreased *Actinomycetales* and *Coriobacteriaceae*, correlating with aggression. Plasma metabolomics revealed that indole and O-acetylcarnitine. butyryl-lcarnitine, 6-hydroxydaidzein, guanidinoacetate, d-fructose, (R)-4-hydroxymandelate, biochanin A, 26-hydroxyecdysone and L-tryptophanwere positively associated with aggression, while kaempferol niacinamide, indican, pyroglutamic acid, 2,4-dinitrophenol, salicylic acid and leucodopachrome were negatively associated. These findings suggest that gut microbiota and plasma metabolites mediate stress-induced aggression in broiler chickens, providing potential targets for managing behavior in stressed poultry.

**Graphical Abstract:**

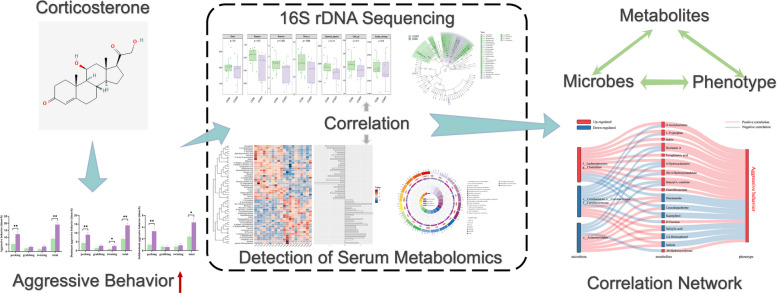

**Supplementary Information:**

The online version contains supplementary material available at 10.1007/s44154-026-00297-2.

## Introduction

Growing concerns over animal welfare in intensive farming practices highlight the need to address the impacts of chronic stress on livestock. Broiler chickens are exposed to various environmental stressors such as temperature (El-Naggar et al. 2019), humidity (Xiong et al. 2017), lighting (Abo-Al-Ela et al. 2021), transportation (Miranda-de la Lama et al. 2018), and stock density (Gomes et al. 2014). Stress is a complex environmental factor that compromises animal health and induces abnormal behaviors, such as aggression (Takahashi 2022; Lupien et al. 2009). Aggressive behavior in poultry can result in economic losses due to reduced productivity and damage to the animals themselves (Bist et al. 2023). Studies have reported that non-beak-trimmed hens exhibit approximately a tenfold increase in mortality due to aggressive behaviors and related injurious pecking (De Haas et al. 2021). Chronic stress activates the hypothalamic–pituitary–adrenal (HPA) axis (Vom Berg-Maurer et al. 2016), increasing glucocorticoid secretion (Cockrem 2013), which influences behavior. However, the exact mechanisms linking stress-induced changes in gut microbiota and plasma metabolites to aggressive behavior remain poorly understood in poultry.

The gut microbiota modulates the host's health and emotional state via bidirectional communication through the microbiota-gut-brain axis, thereby regulating the expression of a wide range of social and emotional behaviors, including aggressive behavior (Bercik et al. 2011; Diaz Heijtz et al. 2011; Cryan and Dinan 2012). Numerous studies have presented that dysregulation of the microbiota-gut-brain axis is associated with abnormal behaviors in laying hens, such as aggressive pecking, feather pecking (FP), and cannibalism (van der Eijk et al. 2019, 2020). Specific microbial products and metabolites can affect the central nervous system and behavior. It is reported that gut microbes have a direct influence on the vagus nerve, resulting in increased c-FOS levels in regions such as the paraventricular and dorsomedial hypothalamic nuclei of the mouse brain, which in turn leads to elevated anxiety-like behaviors (Goehler et al. 2008; Lyte et al. 2006). On the other hand, microbe-produced metabolites and the release of specific immune agonists can also impact behavioral patterns (Morais et al. 2021). For instance, short-chain fatty acids (SCFAs), lipids produced by gut microbiota through the fermentation of dietary fiber, exemplify this mechanism. In preclinical models, SCFAs have been shown to influence the central nervous system by modulating neuroplasticity, epigenetic pathways, gene expression, and immune responses (Silva et al. 2020). Apart from this, gut microbes are capable of synthesizing neurotransmitters or inducing neurotransmitter production in their hosts. Scientific investigations have revealed that multiple microbiota strains, particularly Bacteroides, Bifidobacterium, and Parabacteroides species, possess biosynthetic pathways for gamma-aminobutyric acid production (Strandwitz et al. 2019). Germ-free mice and mice treated with antibiotics exhibit reduced serotonin biosynthesis. However, this reduction can be reversed by inoculating spore-forming bacteria that enhance tryptophan (TRP) metabolism in enterochromaffin cells (Yano et al. 2015). Studies by Hu and colleagues on two different strains of White Plymouth Rock chickens (line 63 and line 72) revealed the following findings: Compared to line 72, line 63 chickens exhibited lower aggression, higher brain levels of 5-hydroxytryptamine (5-HT) and TRP, and a gut microbiome enriched with beneficial bacteria such as *Faecalibacterium* and *Oscillibacter*. These gut microbial profiles were significantly correlated with brain neurotransmitter and plasma hormone levels (Hu et al. 2022). The potential role of compositional changes in intestinal microbiota in mediating stress-related hostile reactions has yet to be conclusively established.

Given the critical role of gut microbiota in regulating emotional and social behaviors, understanding its contribution to stress-induced aggression is crucial. Evidence indicates that changes in the gut microbiota can influence extragut physiological conditions (Karlsson et al. 2013; Sommer and Bäckhed 2013; Tremaroli and Bäckhed 2012). Recent research has shown that changes in serum metabolite profiles are linked to alterations in neurotransmitter concentrations, activation of the HPA axis, and nervous system dysfunction (Sotelo-Orozco et al. 2020; Skalny et al. 2021). Metabolite profile alterations are linked to behavioral changes. Lower blood levels of amino acids like tryptophan and phenylalanine may reduce neurotransmitter concentrations, as these amino acids are neurotransmitter precursors (Kaddurah-Daouk and Krishnan 2009). Zhang et al. found that stress-stressed mice exhibited more anxiety and depression-like behaviors, which were associated with altered tryptophan metabolism (Zhang et al. 2023). Elevating dietary TRP levels in broiler chicken feed has been shown to reduce plasma corticosterone concentrations while increasing 5-HT levels, thereby supporting stress mitigation in intensive poultry farming operations (Bello et al. 2017). Despite growing evidence linking gut microbiota and plasma metabolites to behavior, their specific roles in stress-induced aggression remain unclear.

This study integrates gut microbiota composition and plasma metabolomic data to explore the mechanisms of stress-induced aggression in broiler chickens. By analyzing production performance, hypothalamic serotonin levels, cecal bacterial profiles, plasma metabolic signatures, and aggression scores, we aim to identify key markers associated with aggression under chronic stress. Our findings will provide new strategies for managing stress and improving welfare in poultry farming.

## Results

### Chronic corticosterone exposure reduces growth performance and increases aggressive behavior in broiler chickens

**Fig. 1:**
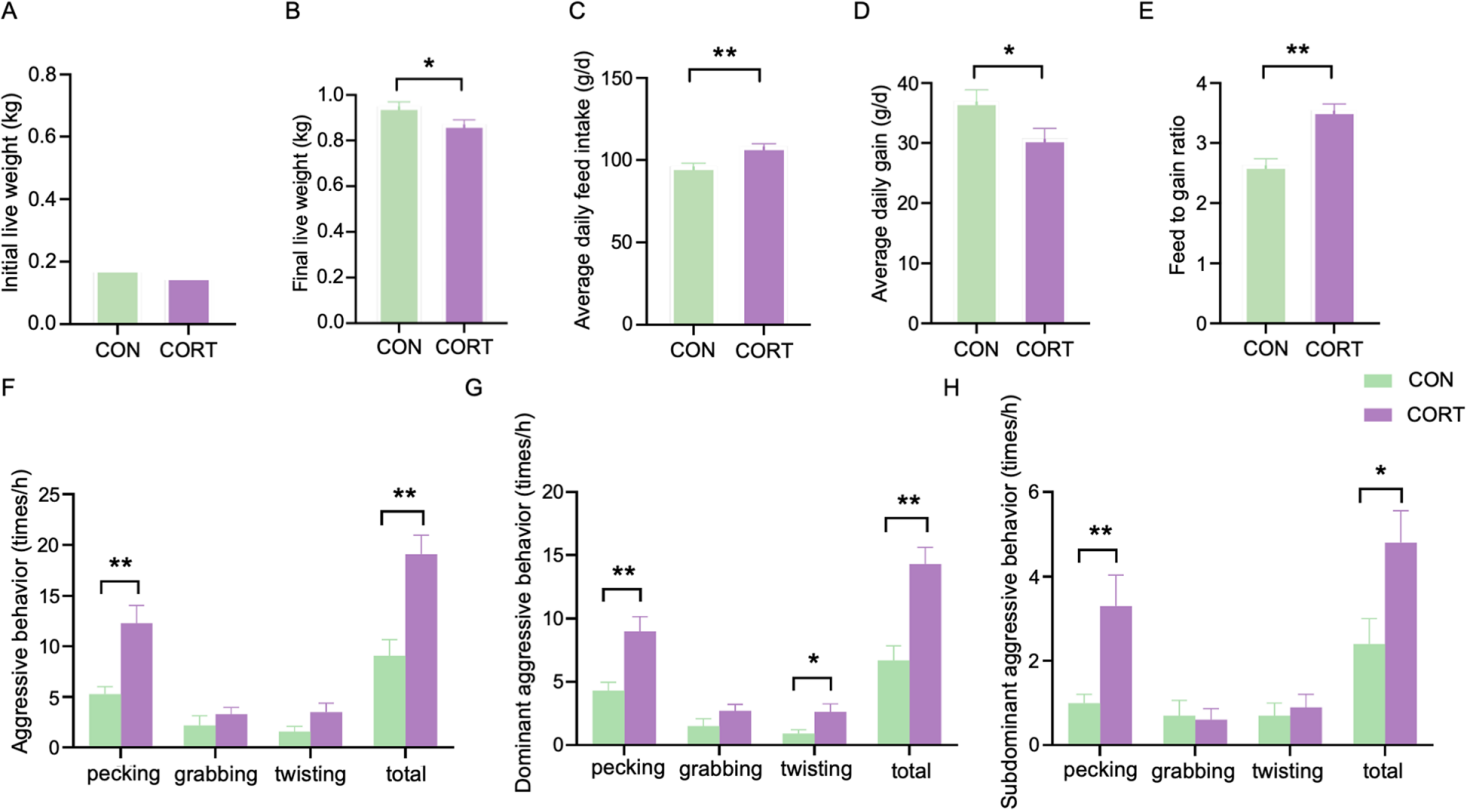
Effect of chronic corticosterone exposure on broiler growth performance and aggressive behavior: **A** Initial weight of broilers at 28 days. **B** final weight of broilers at 35 days. **C** average daily feed intake (ADFI). **D** average daily gain (ADG). **E** feed conversion ratio (F/G). **F** Aggressive behaviors of all broilers. **G** Dominant broilers’ aggressive behavior. **H** Subdominant broilers’ aggressive behavior; *n* = 10, Values are means ± SEM, *P < 0.05, **P < 0.01

Upon commencement of the study, 28-day-old broilers displayed comparable body mass measurements across all treatment groups during initial housing in controlled rearing infrastructure. However, after 35 days, chronic corticosterone exposure significantly impacted growth performance compared to the control group. Specifically, corticosterone-treated broilers exhibited a marked decline in terminal body mass relative to control cohorts (*P* < 0.05; Fig. 1A, B) and average daily gain (*P* < 0.05; Fig. 1D). In contrast, corticosterone treatment markedly increased average daily feed intake (*P* < 0.01; Fig. 1C) and the feed-to-weight ratio (*P* < 0.01; Fig. 1E). As shown in Fig. 1F–H, corticosterone treatment significantly elevated the pecking frequency in broilers, leading to a substantial increase in the total number of aggressive behaviors (*P* < 0.01; Fig. 1F). Among dominant chickens, corticosterone exposure resulted in a significant increase in pecking frequency (*P* < 0.01), twisting frequency (*P* < 0.05), and overall aggressive behavior (*P* < 0.01; Fig. 1G). Similarly, in subdominant chickens, corticosterone-treated birds exhibited significantly higher pecking frequency (*P* < 0.01) and total aggressive behavior (*P* < 0.05; Fig. 1H). Although corticosterone exposure did not significantly alter hippocampal serotonin concentrations (Supplementary Table 2), it significantly affected serotonin levels in both plasma and the hypothalamus. Plasma serotonin concentrations increased (*P* < 0.05), while hypothalamic serotonin levels decreased (*P* < 0.01; Supplementary Table 2). On the other hand, there were no significant changes in dopamine levels in plasma, hippocampus, or hypothalamus following corticosterone exposure.

### Chronic corticosterone exposure alters the cecal microbiota of broiler chickens

**Fig. 2:**
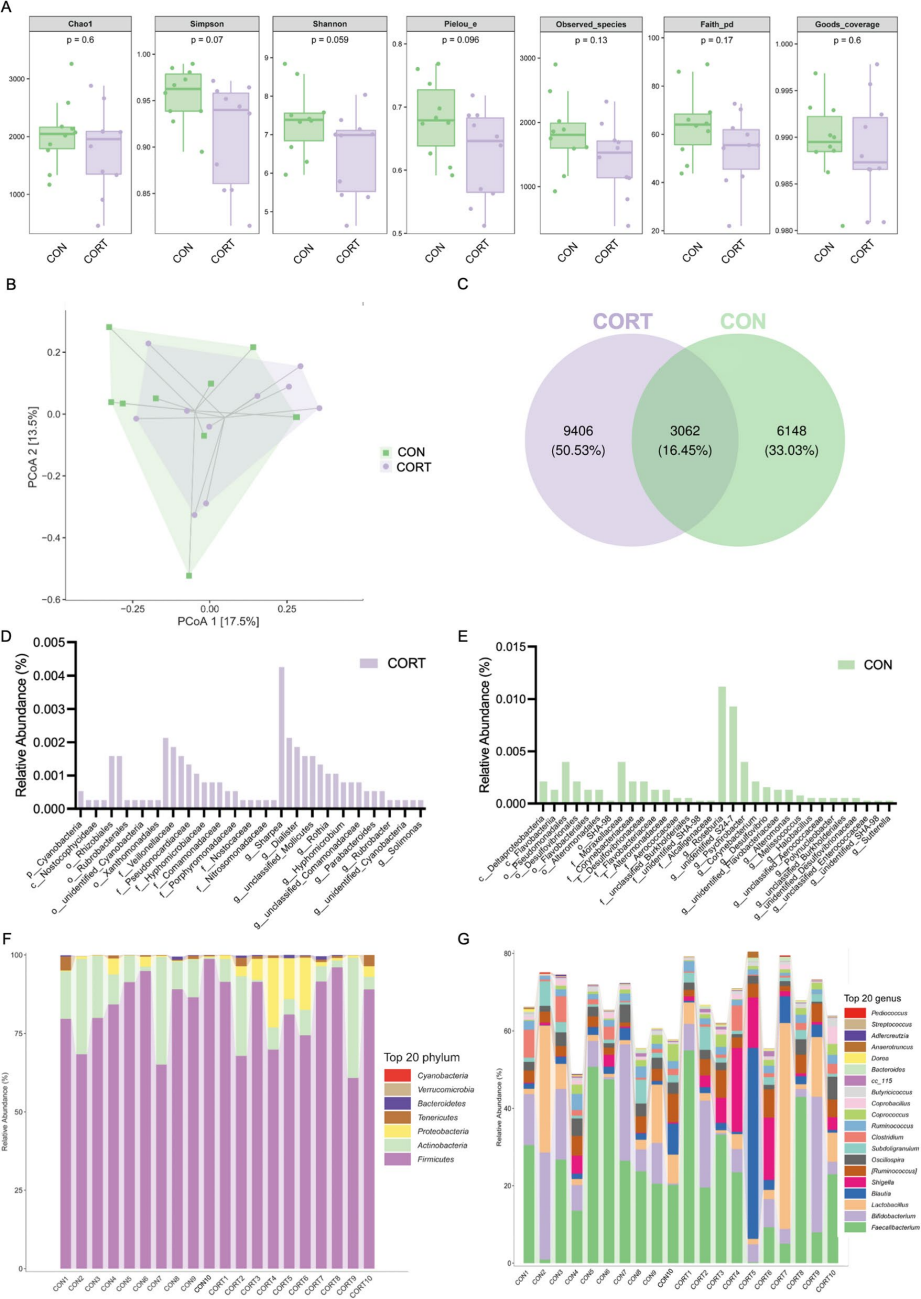
Impacts of chronic corticosterone exposure on the cecal microbiota diversity and composition in broilers: **A** Cecal content microbial α-diversity. **B** Beta diversity (determined by principal coordinate analysis (PCoA)) of gut microbial community based on OTUs abundance between the two groups. **C** Venn diagram of the distribution of OTUs among different groups. **D** The relative abundance of uniquely expressed microbiota in CORT group. **E** The relative abundance of uniquely expressed microbiota in CON group. **F** Relative abundance of the cecal content microbiota in the phylum. **G** Relative abundance of the cecal content microbiota in the genus

Twenty fecal specimens comprising 52,895 high-quality sequences underwent operational taxonomic unit (OTU) clustering analysis. Seven α-diversity metrics (Chao1, Simpson, Shannon, Pielou_e, Observed_species, Faith_pd, and Goods_coverage) were calculated to evaluate diversity and richness within intestinal microbial communities. These indices showed no significant differences between the control (CON) and corticosterone-treated (CORT) groups (*P* > 0.05; Fig. 2A). The Goods_coverage index was above 98% for all groups, confirming that the majority of microbial diversity had been captured in the current study (Fig. 2A). To evaluate the structural differences in gut microbiota, β-diversity analyses were performed. Principal coordinate analysis (PCoA) based on Bray–Curtis distances revealed that the microbial community structures in the feces of corticosterone-treated and control broilers were similar (Fig. 2B). A Venn diagram illustrated the distribution of OTU counts, highlighting unique and shared OTUs between the two groups (Fig. 2C). Furthermore, the relative abundance of microbial taxa unique to the CON and CORT groups was visualized (Fig. 2D, E). Firmicutes and Actinobacteria were identified as the predominant phyla in both groups (Fig. 2F). At the genus level, *Faecalibacterium, Bifidobacterium*, and *Lactobacillus* were the dominant genera in both groups (Fig. 2G).

**Fig. 3:**
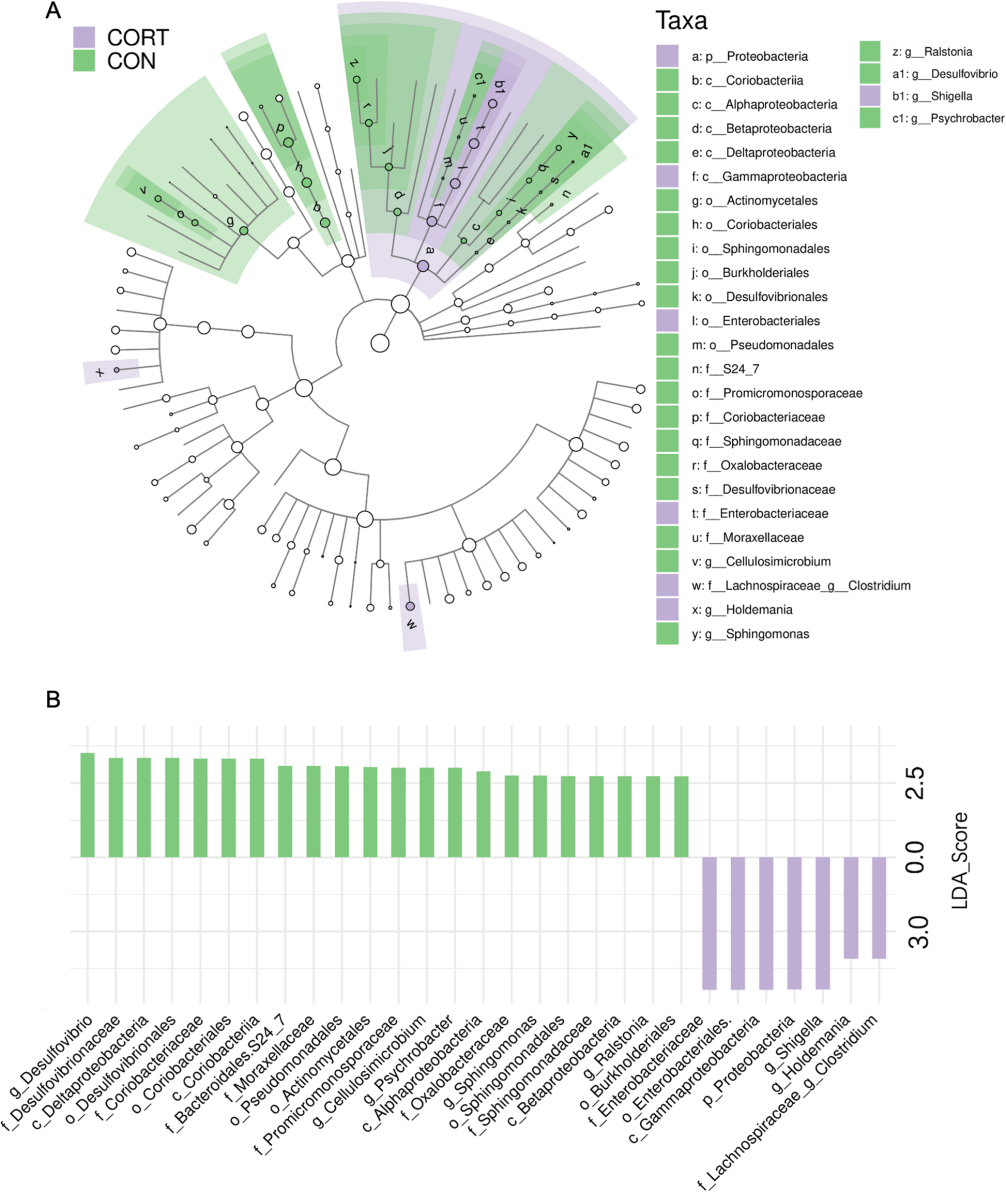
Effect of chronic corticosterone exposure on differential cecal flora in broiler chickens. **A** Cladogram representation of the differentially abundant taxa among two groups; **B** Identification of differential bacterial taxa by LefSe tool (LDA score > 2.0, *P* < 0.05)

To identify specific bacterial taxa affected by corticosterone, linear discriminant analysis effect size (LEfSe) analysis was conducted. Bacterial taxa with *P* < 0.05 and LDA > 2.0 were considered significant, resulting in the identification of 29 differentially abundant taxa (*P* < 0.05; Supplementary Table 2). Phylum-level analysis indicated a marked rise in Proteobacteria prevalence among CORT-exposed birds. Genera *Shigella*, *Holdemania*, and *Clostridium* displayed notable upregulation in CORT samples, whereas *Desulfovibrio*, *Cellulosimicrobium*, and *Enterococcus* populations exhibited prominent declines relative to CON controls. Additionally, at other taxonomic levels, the abundance of *Coriobacteriaceae* and *Bacteroidales.S24_7* was significantly lower in corticosterone-treated chickens (Fig. 3A, B).

### Chronic corticosterone exposure changes metabolic profiles of plasma in broiler chickens

**Fig. 4:**
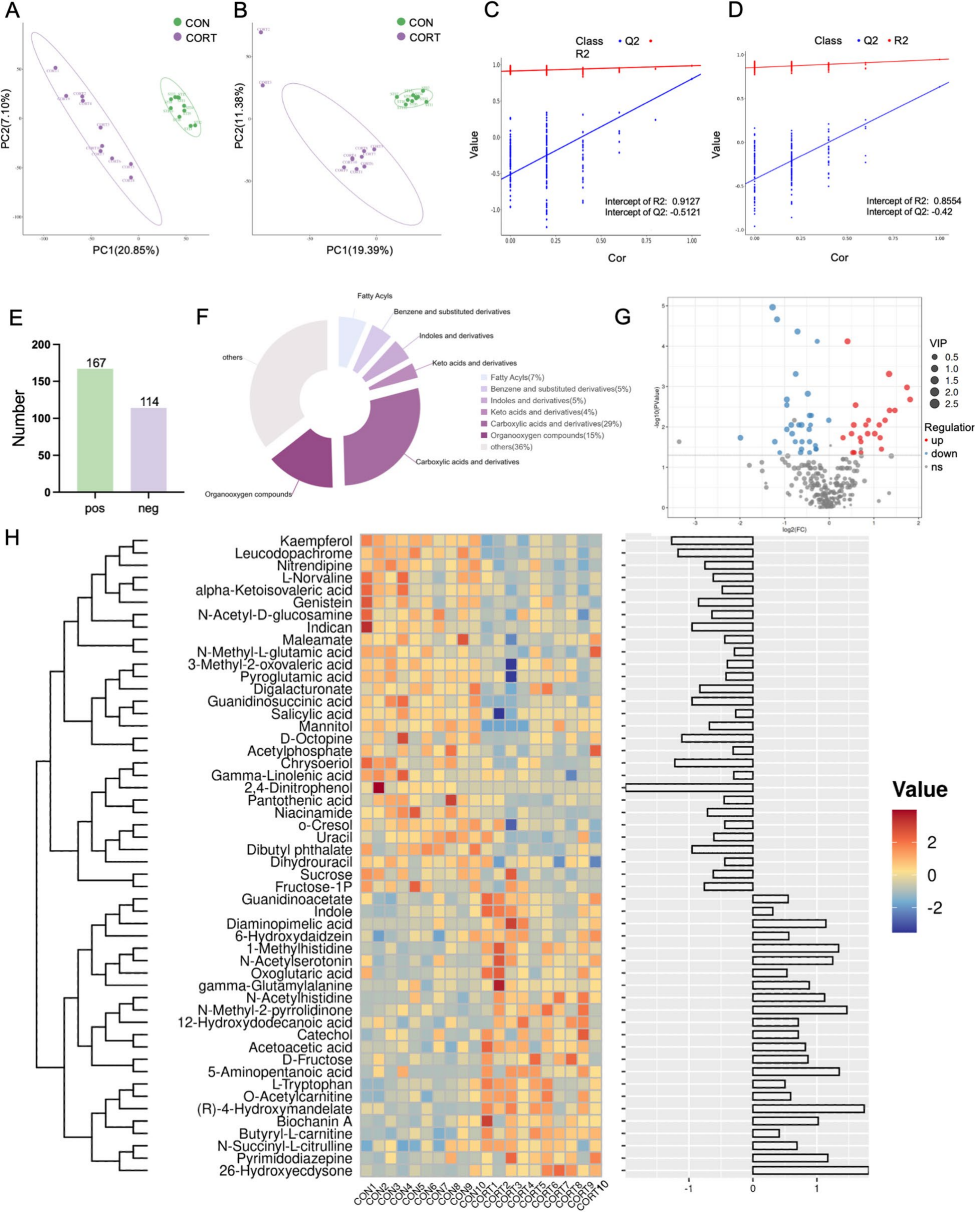
Effects of corticosterone on metabolic profiles of plasma in broilers. **A** Partial Least Squares-Discriiminate Analysis (PLS-DA) score plot of metabolites in positive mode; **B** Partial Least Squares-Discriiminate Analysis (PLS-DA) score plot of metabolites in negative mode; **C** Permutation test plot of PLS-DA score plot in positive mode; **C** Permutation test plot of PLS-DA score plot in negative mode; **E** Numbers of metabolites in different groups; **F** Distribution of the relative abundance of metabolites of each Class in different groups; **G** Volcano plots of differential metabolites; **G** Heat map of differential metabolites

To assess the impact of corticosterone on plasma metabolites, a metabolomics analysis was performed. Partial least squares discriminant analysis (PLS-DA) revealed distinct metabolic profiles between corticosterone-treated and control broilers (Fig. 4A–D). A total of 281 metabolites were identified, including carboxylic acids and their derivatives (23.57%), indoles and their derivatives (4.29%), and benzene and its substituted derivatives (4.29%) (Fig. 4E, F). A total of 53 metabolites exhibited altered abundance levels between experimental cohorts, with 23 displaying significant elevation and 30 showing reduction specifically in the CORT group relative to controls (Fig. 4G). A heatmap of these metabolites further highlighted the distinct metabolic patterns between groups (Fig. 4H).

**Fig. 5:**
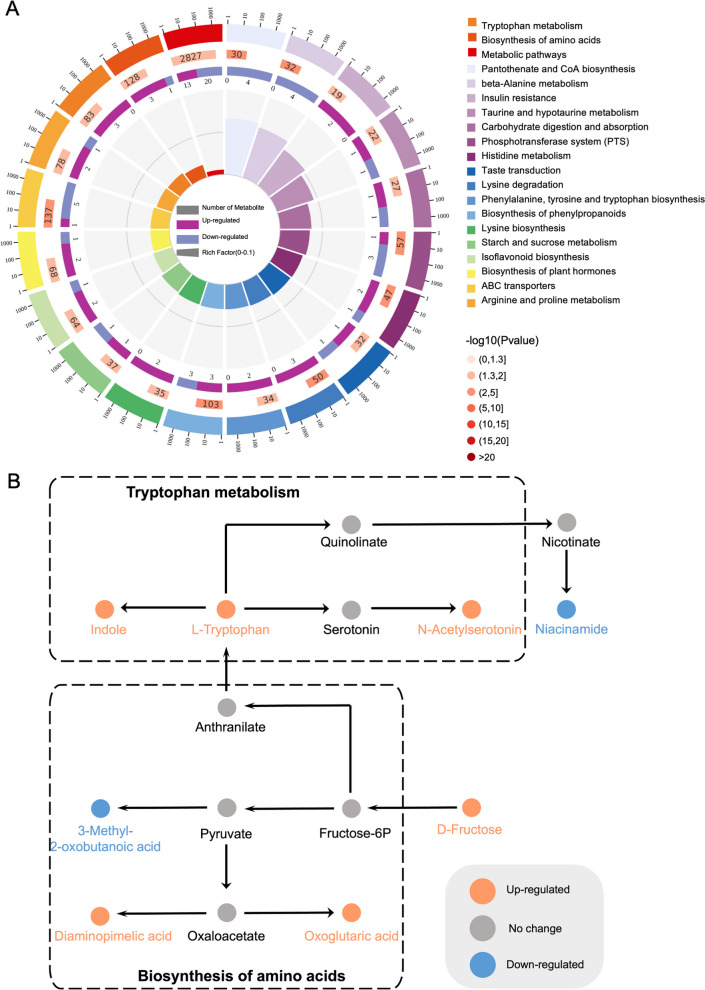
Effect of corticosterone exposure on the plasma differential metabolites related pathways in broiler chickens. KEGG term analysis of metabolites related pathways in broiler chickens. There are four circles from outside to inside in (**A**). The first circle: the classification of enrichment; outside the circle is the coordinate ruler of the number of metabolites. The second circle: the number of the classification in the background metabolite and the *P*-value. The more metabolites, the longer the bar; the smaller the *P*-value, the deeper the color. The third circle: the bar graph of metabolites. The fourth circle: the rich factor value of each category. **B** Metabolic pathway networks responding to CORT exposure

Pathway enrichment analysis mapped these metabolites onto 25 KEGG metabolic pathways (*P* < 0.05; Table S3). The most significantly affected pathways included pantothenate and CoA biosynthesis, beta-alanine metabolism, and the biosynthesis of phenylpropanoids (Fig. 5A). Tryptophan metabolism was highlighted as a key pathway associated with corticosterone exposure, with tryptophan and its derivatives upregulated (Fig. 5B). These results suggest that corticosterone-induced stress disrupts the plasma metabolome in broiler chickens and highlights specific metabolic pathways that may influence behavior.

### Potential associations among the gut microbiome, plasma metabolites, and aggressive behavior in broiler chickens after chronic corticosterone exposure

**Fig. 6:**
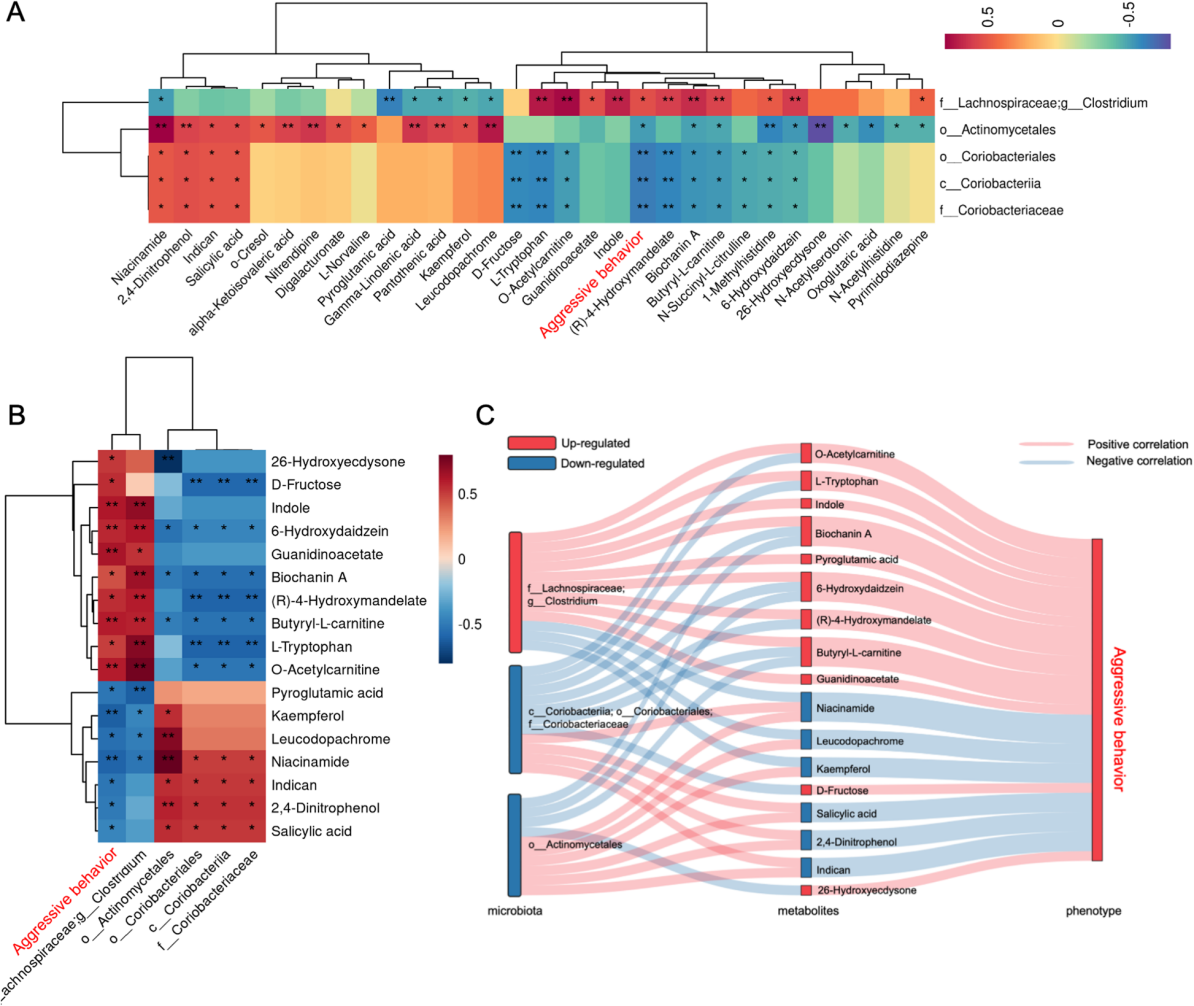
Association between differential metabolites, differential metabolites and phenotype. Spearman correlations between differential bacteria and differential metabolites and aggressive behavior; **A** Illustrates all the flora that are significantly associated with aggressive behavior; **B** Illustrates all the metabolites that are significantly associated with aggressive behavior; **C** Interrelationship between gut microbiota composition, host metabolic profile and aggressive behavior phenotype, the asterisks (*) in correlation heatmaps indicate *P*-value < 0.05, (**) in correlation heatmaps indicate *P*-value < 0.01

To explore the relationships among gut microbiota, plasma metabolites, and aggressive behavior, Spearman’s rank correlation analyses were conducted. First, correlations between gut microbial genera and aggressive behavior were examined. The results revealed a positive correlation between aggressive behavior and *Clostridium*, and a negative correlation with *Actinomycetales* and *Coriobacteriaceae*. Next, the correlations between differentially abundant gut microbiota and plasma metabolites were analyzed. A total of 31 metabolites showed significant correlations. For example, *Clostridium* positively correlated with indole, L-tryptophan, and butyryl-L-carnitine, but negatively correlated with niacinamide, pantothenic acid, and leucodopachrome. Conversely, *Actinomycetales* positively correlated with niacinamide and gamma-linolenic acid while negatively correlated with 26-hydroxyecdysone and 1-methylhistidine. Similarly, *Coriobacteriaceae* positively correlated with indicant, niacinamide, and salicylic acid but negatively correlated with d-fructose and butyryl-L-carnitine (Fig. 6A). Lastly, the association between aggressive behavior and plasma metabolites was analyzed. Aggressive behavior was positively associated with metabolites such as indole, (R)−4-hydroxymandelate, and L-tryptophan, but negatively associated with niacinamide, leucodopachrome, and pyroglutamic acid (Fig. 6B). By integrating these relationships, a Sankey diagram was constructed to link gut microbiota, plasma metabolites, and behavioral phenotypes (Fig. 6C), providing a comprehensive view of their interactions.

## Discussion

Multi-omics strategies are increasingly adopted to dissect the regulatory mechanisms of complex diseases and traits (Weng et al. 2024), yet few studies have explored aggressive behavior in stressed broilers. Our findings reveal that chronic corticosterone exposure alters cecal microbiota, modifies plasma metabolite profiles, and heightens aggressive behavior. *Clostridium*, Coriobacteriaceae, and Actinomycetales potentially play an important role in inducing aggressive behavior, a link not previously described. Tryptophan and its derivatives also show significant associations with aggression, suggesting a biochemical pathway.

Corticosterone injections are a standard method to simulate chronic stress in poultry, as shown in prior studies (Hu et al. 2018; Lin et al. 2006; Zaytsoff et al. 2022; Zulkifli et al. 2014). In this work, Yellow-feather broilers were given corticosterone by subcutaneous injection for seven consecutive days to simulate chronic stress. Several studies have investigated the effects of corticosterone on feed intake and body weight in poultry, with results varying across studies. In 24-week-old Hy-line brown poultry subjects, the subcutaneous delivery of corticosterone at 2 mg/kg over 7 days induced definitively measurable declines in body mass while feed consumption also decreased proportionally, as reported by Liu et al.'s study (Liu et al. 2012). Yuan et al. used Arbor Acres male broilers and supplemented their diet with corticosterone at 30 mg/kg of diet. In one-week-old broilers treated for 7 days, corticosterone significantly inhibited weight gain, substantially increased feed intake, and significantly lowered the feed-to-gain ratio. Similar results were also observed in four-week-old broilers treated for 6 days (Yuan et al. 2008). Hu et al. also adopted the same treatment method by adding corticosterone to the feed of Arbor Acres one-day-old broilers. The results indicated that while the final body weight and daily weight gain of the broilers were reduced, feed intake remained unchanged. This mechanism was attributed to the activation of the hypothalamic LKB1-AMPK-NPY/ACC signaling pathway (Hu et al. 2020). Our experimental results were consistent with those reported by Yuan et al. Chronic corticosterone exposure significantly reduced the final body weight and average daily weight gain of broilers, while markedly increasing feed intake and the feed conversion ratio. This may reflect increased energy use and a shift towards fat deposition, possibly driven by proteolysis and gluconeogenesis, highlighting stress’s metabolic impact.

Aggressive behavior in chickens is a multifaceted social trait, and our study confirms that chronic stress amplifies it in both dominant and subdominant individuals (as shown in Fig. 1F-H). Within socio-ecological contexts, heterogeneous adaptive capacities exist across individuals to adjust physiological and behavioral traits—particularly personality dimensions like locomotor activity and aggressive behavior—when responding to ecologically distinct stressors, reflecting interindividual divergence in adaptive plasticity (Biro and Stamps 2008; Moore and Martin 2019; Chunduri et al. 2022). Based on a dominance hierarchy or a ranking order, subdominant individuals manifest aversive fear responses toward conspecific dominants, consequently compromising their phenotypic plasticity in adapting to captivity-regulated environmental parameters. Conversely, dominant cohorts show neurobiologically reinforced aggression escalation—marked by heightened incidence of injurious pecking behaviors—mediated through activation of mesolimbic reward circuitry and operant conditioning mechanisms (Banich and Floresco 2019). Dominant birds exhibit more aggression, likely reinforced by neural reward pathways, consistent with earlier findings. This aligns with Ahmed et al.’s observation of increased aggression from embryonic corticosterone exposure (Ahmed et al. 2020) and El-Lethey et al.’s findings on social stress in hens (El-Lethey et al. 2000). Our data validate that stress not only impairs growth but also induces behavioral shifts, particularly increased aggression.

The microbiota-gut-brain axis plays a critical role in modulating brain function and behavior. (Bercik et al. 2011; Cryan and Dinan 2012). Disruption of the gut microbiota can lead to various intestinal diseases and impair brain function, potentially resulting in neuropsychiatric disorders characterized by abnormal behavior (Bioque et al. 2021; Cryan and O'Mahony 2011). Our analysis showed no significant α-diversity change in cecal microbiota between control and stressed broilers, though a decreasing trend was noted in the stressed group, with similar β-diversity. At the phylum level, *Firmicutes* and *Actinobacteria* were the dominant bacterial phyla, while at the genus level, the predominant genera included *Faecalibacterium*, *Bifidobacterium*, and *Lactobacillus*. This observation aligns with previous studies on the composition of the chicken gut microbiota (Wei et al. 2013). Using LDA Effect Size, we found increased *Shigella*, *Holdemania* and *Clostridium*, and decreased *Bacteroidales.S24_7*, Coriobacteriaceae, *Desulfovibrio*, Psychrobacter, Cellulosimicrobium, Sphingomonas and Ralstonia in stressed birds. Notably, *Clostridium*, Coriobacteriaceae and Actinomycetales were significantly associated with aggressive behaviors (*P* < 0.05, Spearman's ρ = 0.53, −0.63, −0.52). Currently, limited research has explored the link between the three aforementioned bacterial taxa and aggressive behavior. Barandouzi et al.'s systematic review mentions that in patients with depression, the abundance of *Clostridium XIX* and *IV* is significantly increased, indicating their potential role in mood regulation (Barandouzi et al. 2020). Furthermore, bacteria within the family Coriobacteriaceae (specifically the butyrate-producing genera *Eggerthella* and *Atopobium*) were found to exhibit increased relative abundance in depressed individuals (Barandouzi et al. 2020). In cases of aggressive periodontitis, the abundance of bacteria within the order Actinomycetales, particularly *Aggregatibacter actinomycetemcomitans*, has been significantly elevated (Montenegro et al. 2020). Increased Clostridium is linked to depression, and certain Actinomycetales to aggressive periodontitis, though direct links need to be further explored.

Although a standard cage-rearing system and a commercial, standardized broiler diet were used to minimize variation attributable to housing and feed, environmental factors may still influence gut microbiota and behavior. For instance, diet composition, including fiber content and feed form, modulates microbial fermentation and metabolic outputs (Mahmood et al. 2020; Bindari et al. 2022); changes in housing systems and the rearing environment can reshape community structure independent of diet composition (Kers et al. 2018). Additionally, lighting regimen and intensity affect broiler activity, social interactions, and welfare (Wu et al. 2022; Olanrewaju et al. 2016); and stocking density is associated with performance, stress physiology, and behavioral outcomes (Li et al. 2022). Therefore, the specific taxa–metabolite–behavior links observed here should be validated under alternative production conditions, such as floor-pen rearing, variable stocking densities, different lighting programs, and varied diets, to enhance generalizability.

Given gut microbiota’s role in metabolism, we examined how corticosterone-induced shifts affect plasma metabolites and behavior. Untargeted metabolomics showed clear group separation via PLS-DA, with chronic stress altering metabolite profiles. Compared to the control group, 23 metabolites were upregulated, while 30 were downregulated. KEGG enrichment analysis revealed that differential metabolites were primarily involved in tryptophan metabolism and pantothenate-CoA biosynthesis, pathways closely linked to serotonergic signaling and aggression. Subsequent correlation analysis identified 31 metabolites associated with the three differential microbes that had been previously identified. Further analysis revealed a correlation between the levels of specific metabolites and changes in aggressive behavior following corticosterone exposure. Of the 31 differential metabolites, 18 were found to have correlation with aggressive behavior, including indole, niacinamide, L-tryptophan, biochanin A, and so forth. Notably, among these screened differential metabolites, tryptophan (Trp) and its derivatives merit particular attention. The rationale resides in the plausible mechanism whereby gut microbiota influences mental health through modulation of tryptophan and serotonin (5-hydroxytryptamine, 5-HT) metabolism.

The central 5-hydroxytryptamine (5-HT) system plays a critical role in regulating personality traits such as depression, aggression, impulsivity, and anger in humans and animals (Zimmermann et al. 2012; Coccaro et al. 2015). The raphe nuclei are the principal source of central 5-HT synthesis, whereas > 90% of total body 5-HT is produced by intestinal enterochromaffin (EC) cells; importantly, peripheral 5-HT does not cross the blood–brain barrier (BBB) (Yano et al. 2015). Enhanced central serotonergic activity is associated with behavioral inhibition, particularly suppression of aggression (da Cunha-Bang et al. 2016; Wolkers et al. 2017), whereas diminished central serotonergic activity correlates with heightened aggression and impulsivity across species, including mammals, birds, fish, and rodents (Bannai et al. 2007; Sperry et al. 2003; Clotfelter et al. 2007; Liu et al. 2019). Supporting this, our CORT regimen significantly reduced hypothalamic 5-HT, consistent with prior observations (Ahmed et al. 2014). Furthermore, Elevated circulating 5-HT has also been reported in aggressive laying-hen strains and in humans with aggression-linked psychopathology (Bello et al. 2017). Peripheral 5-HT cannot permeate the BBB, its behavioral associations likely operate via peripheral–central signaling routes rather than by direct entry into the brain. In our cohort, plasma 5-HT increased concomitantly with higher plasma tryptophan, while central 5-HT decreased—a pattern compatible with enhanced peripheral 5-HT synthesis or altered platelet sequestration secondary to greater tryptophan availability. EC-cell–derived 5-HT serves as a key signal for vagal afferents (Cao et al. 2021), and peripheral 5-HT synthesis is shaped by the gut microbiota (Jadhav et al. 2022). Accordingly, CORT-induced microbial shifts may alter EC-cell 5-HT output and, via the vagus nerve, reconfigure signals relayed to the brain. In addition, gut bacteria such as members of the genus Clostridium metabolize tryptophan into neuroactive compounds (e.g., indoles and tryptamines) that can stimulate vagal afferents (Kaur et al. 2019). This neuronally mediated gut–brain pathway could contribute to the observed reduction in hypothalamic 5-HT and heightened aggression under chronic stress.

Beyond direct neural signaling, the gut microbiota can profoundly affect brain function and behavior through the host immune system (Gao et al. 2018). The kynurenine pathway (KP) degrades most peripheral tryptophan (> 90%) and is highly responsive to immune activation (Kaur et al. 2019). Chronic stress and dysbiosis elicit low-grade systemic inflammation, promote pro-inflammatory cytokine release, and activate the pathway’s rate-limiting enzymes—indoleamine 2,3-dioxygenase (IDO) and tryptophan 2,3-dioxygenase (TDO) (Höglund et al. 2019). In this study, higher plasma tryptophan concomitant with reduced hypothalamic 5-HT suggests that, under chronic stress, the overall tryptophan pool may expand while its metabolic routing shifts. Activation of the KP diverts a larger proportion of tryptophan away from 5-HT biosynthesis, thereby reducing the fraction of tryptophan that crosses the BBB via LAT1 to serve as the precursor for central 5-HT (Kaur et al. 2019). Moreover, KP metabolites such as kynurenine and quinolinic acid are intrinsically neuroactive and have been implicated in the pathophysiology of behavioral disorders, including depression (Roth et al. 2021). Notably, microbial metabolites directly engage immune signaling: the aryl hydrocarbon receptor (AhR), expressed by intestinal immune cells, is a key regulator of gut homeostasis. Both kynurenine and indole derivatives produced by gut bacteria, including Clostridium spp., are established AhR ligands (Gao et al. 2018). In the CORT group, microbiota alterations may increase these ligands, modulate intestinal immune tone and foster an inflammatory environment that biases tryptophan toward the KP, thereby diminishing central 5-HT synthesis and contributing to heightened aggression. Together with vagal signaling, this immune–metabolic mechanism links chronic stress and dysbiosis to serotonergic deficits and aggressive behavior.

Our multi-omics data indicate that chronic corticosterone stress in broilers increases aggression while altering cecal taxa and circulating metabolites, with Trp metabolism and central 5-HT emerging as key nodes. These patterns suggest several nutrition-based levers. First, ensuring adequate dietary Trp and an appropriate Trp:Lys ratio can support serotonergic tone and reduce abnormal pecking. Studies have shown that increasing dietary Trp or its precursor 5-hydroxytryptophan reduces fearfulness and gentle feather pecking, while acute Trp depletion has the opposite effect; chronic Trp supplementation decreases such behaviors (Birkl et al. 2019; Lundgren et al. 2023). In commercial nutrition programs, target Trp:Lys ratios around 16–19% are widely referenced and align with improving behavior and performance (Linh et al. 2021). Second, probiotics that act along the microbiota–gut–brain axis can mitigate stress-linked injurious pecking. Randomized studies in chickens report that L. rhamnosus reduces stress-induced feather pecking, and Bacillus subtilis decreases injurious behavior while modulating the gut–brain axis (Mindus et al. 2021). Beyond live microbes, sodium butyrate (a microbial postbiotic) counters corticosterone-induced oxidative stress and helps stabilize intestinal function under environmental stressors, supporting a calmer behavioral phenotype (Zhang et al. 2011). In conclusion, the multi-omics evidence presented provides a theoretical foundation for exploring the potential efficacy of supplementing tryptophan and utilizing microbial and postbiotic additives to enhance behavior.

This study proposes a novel integrative framework linking gut microbiota, plasma metabolites, and aggressive behavior, underscoring the central role of tryptophan metabolism and its derivatives. Specifically, chronic corticosterone exposure may alter the composition of gut microbiota, modulating circulating tryptophan levels, affecting brain 5-HT synthesis, and ultimately exacerbating aggressive behavior in broilers. Our findings identified three key gut microbial genera and 18 metabolites, with tryptophan and its derivatives occupying a central role within this network. While our multi-omics analyses implicate specific taxa and Trp-linked metabolites in stress-related aggression, association alone does not establish causation. Convergent evidence from germ-free and fecal microbiota transplantation (FMT) models in mammals shows that gut microbial communities can transfer behavioral phenotypes, including anxiety-like and depressive-like traits, to recipients (Bercik et al. 2011; Zheng et al. 2016). Moreover, FMT from donors exhibiting altered stress responses has been sufficient to induce similar behavioral patterns in recipients (Kelly et al. 2016), indicating that microbiota-encoded functions can influence brain and behavior. Mechanistically, spore-forming Clostridiales have been shown to promote host enterochromaffin cell 5-HT biosynthesis (Yano et al. 2015), and species like Ruminococcus gnavus and Clostridium sporogenes, can metabolize Trp into neuroactive compounds such as tryptamine and indole-3-propionic acid (Williams et al. 2014). These influence serotonergic pathways, linking microbial Trp metabolism to central 5-HT and aggression. To establish causation in chickens, future studies could use FMT from donors with differing stress phenotypes, gnotobiotic or defined-consortia colonization to isolate taxa effects, and targeted Trp manipulations. Despite these insights, knowledge of gut microbiota dynamics under chronic stress and its effects on host metabolism and behavior remains limited. Future research should employ targeted metabolomics and microbiota transplantation to validate these links, while exploring temporal dynamics for deeper understanding.

## Conclusions

In summary, chronic stress impairs broiler growth, lowers central serotonin, and increases aggression, with changes in gut microbiota and plasma metabolites. Tryptophan metabolism is central, offering a foundation for further studies and potential interventions to manage stress-induced behavioral disorders in poultry.

## Methods and materials

### Animals and experimental design

Fifty yellow-feather broilers chickens (one-day-old) were selected for the experiment and housed in standard cages (size 90 cm*60 cm*40 cm) within a temperature-controlled animal room at Nanjing Agricultural University. The chickens were subjected to a 16-h light/8-h dark daily light cycle. The chicks were fed a commercial broiler diet as recommended by the NRC (1994). At 28 days, chickens were grouped into body weight-matched pairs and randomly assigned to either a control group or a corticosterone-treated group. Chickens assigned to the corticosterone-treated group received once daily subcutaneously injected corticosterone with a dosage of 4 mg/kg body weight. (between 13:00 and 15:30). The control group was administered a subcutaneous injection of 15% ethanol solution at the same dosage as the corticosterone group. The treatment lasted seven consecutive days, during which a chronic stress model was established. Food and water were freely available throughout the trial period. Body weight and feed intake were recorded daily. Corticosterone was procured from J&K Scientific. It was initially dissolved in 100% ethanol to a concentration of 1% as a stock solution, maintained in light-protected conditions at 4 °C. For experimental use, aliquots were diluted with sterile saline to achieve final ethanol content reduced to 15% (v/v).

### Aggression experiment

Behavioral evaluations for aggression commenced during days 35–36 post-hatching. A dedicated testing chamber physically separated from the poultry rearing environment contained dimensionally standardized enclosures (90 cm × 60 cm × 40 cm), mirroring the containment systems employed during broiler development for experimental assessment.

According to the prescribed protocol, experimental cohorts underwent randomized sampling (*n* = 100/group) with subsequent anatomical differentiation using dermatologically applied chromatic discriminators ensuring spatial exclusivity across integration zones. Two chickens from the same group but different cages (with no prior contact) were concurrently introduced into the experimental arena. Interactions were video-recorded for 60 min before transitioning to the next experimental pair. During the recording, the experimenters maintained a distance of at least 1 m from the test area. Four synchronized cameras, connected to a computer, were mounted above the four test pens. Behavioral data were recorded and subsequently transferred to the laboratory for analysis. The daily test sessions were scheduled as follows: 8:00–11:00 and 14:00–17:00, conducted over two consecutive days.

Following data collection, three independent assessors blinded to the experimental groups reviewed and analyzed the footage, quantifying the frequency of aggressive behaviors exhibited by each group of broilers over the 60-min observation period. Aggressive behavior categories were categorized according to the methodology outlined by Kitaysky (2003), as detailed in Table S1. Additionally, the dominant and subordinate broilers in each group were determined using Froman's method, which assesses the social dominance between paired broilers based on how often one broiler avoids another during the aggressive behavior test (Froman et al. 2002). The broiler that is clearly avoided is considered the dominant broiler.

### Samples collection and analysis

On day 37, following a 12-h fasting period, Yellow-feather broilers were weighed and subsequently euthanized via decapitation. Blood samples were immediately collected, treated with anticoagulant, and placed on ice for two hours. Plasma was then obtained by centrifugation at 3500 rpm for 10 min. The resulting plasma was aliquoted into cryogenic vials and stored at −20 °C pending metabolomic analysis. Cecal specimens were aseptically harvested from poultry subjects, immediately flash-frozen in liquid nitrogen, and subsequently transferred to −80 °C storage for downstream analysis.

### Determination of 5-HT content

The chicken serotonin ELISA kit (MM-204101) and chicken dopamine ELISA kit (MM-6004401), purchased from Jiangsu Meibiao Biotechnology Co., Ltd, were utilized to quantify serotonin and dopamine concentrations in plasma and brain tissues (hippocampus, hypothalamus) of broilers. Experimental analyses adhered strictly to protocols during procedural execution.

### 16S rRNA sequencing of cecal contents

Total genomic DNA was extracted from cecal content using a commercial DNA extraction kit. DNA concentration and purity were assessed using a NanoDrop spectrophotometer (Thermo Fisher Scientific, USA), and quality was verified via 1.2% agarose gel electrophoresis. The V3-V4 region of the bacterial 16S rRNA gene was amplified using PCR with primers containing sample-specific barcodes:

- Forward Primer: 5′-ACTCCTACGGGAGGCAGCA-3′
- Reverse Primer: 5′-GGACTACHVGGGTWTCTAAT-3′

PCR reactions employed Pfu Ultra High-Fidelity DNA Polymerase (TransGen Biotech, China) under standard cycling conditions. Amplified products (25 μL) were purified with Vazyme VAHTSTM DNA Clean Beads at a 0.8 × bead-to-sample volume ratio. Purified DNA was quantified fluorometrically using the Quant-iT PicoGreen dsDNA Assay Kit (Thermo Fisher Scientific) on a BioTek FLx800 microplate reader (Agilent Technologies, USA).

### Microbiome data processing and analysis

The preparation of sequencing libraries was conducted using Illumina's TruSeq Nano DNA LT Library Prep Kit, while community DNA fragments were subjected to sequencing utilising the Illumina MiSeq/NovaSeq platform with paired-end sequencing. Raw sequencing reads were stored in FASTQ format and subjected to denoising and error-correction steps using the DADA2 algorithm. Sequence clustering was performed to generate operational taxonomic unit (OTU), and subsequent taxonomic annotation was executed via alignment with the Greengenes database for microbial species identification. The calculation of sample diversity was carried out using QIIME2 software, and the inter-group differences (β-diversity) were analyzed based on the Bray–Curtis PCoA algorithm, with PERMANOVA tests for validation. Linear discriminant analysis with effect size (LEfSe) was then used to differentiate between microbes in chronic corticosterone exposure groups and control groups. Functional predictions were made by normalizing the OTU abundance table with PICRUSt and aligning sequenced genes with the MetaCyc database for functional annotation.

The denoising and clustering of sequences was performed using the DADA2 method, with each dereplicated sequence post-DADA2 quality control referred to as an OTU. The Greengenes database was used for the annotation of species. The assessment of alpha diversity was conducted using QIIME2 (2019.4) software to calculate Simpson's index, Shannon's index, observed species, and Chao1 index. Inter-group differences were analysed based on the Bray–Curtis PCoA algorithm, with *P*-values calculated using PERMANOVA tests to assess β-diversity. The R package (Python LEfSe package, R ggtree, ggplot2 package) was utilized to implement LEfSe, a methodology that integrates linear discriminant analysis with effect size, to select microbes with LDA > 2 and *P* < 0.05 (*P*-values calculated using Wilcoxon rank-sum tests, and LDA values calculated using Linear discriminant analysis).

Functional prediction analysis was performed according to the following workflow: The OTU abundance table was normalized using PICRUSt for subsequent analyses. The obtained gene sequences were then aligned against the MetaCyc database for comprehensive functional annotation.

### LC–MS analysis of plasma

Plasma samples were thawed at 4 °C and vortexed for 1 min to ensure homogeneity. A 100 μL aliquot of each sample was precisely pipetted into a 2 mL centrifuge tube. Subsequently, 400 μL of methanol solution (previously stored at −20 °C) was added, and the mixture was vortexed for 1 min. The samples were then centrifuged at 12,000 rpm and 4 °C for 10 min. The entire supernatant was carefully transferred to a fresh 2 mL centrifuge tube and dried under concentration. For reconstitution, exactly 150 μL of an 80% methanol aqueous solution containing 2-chloro-L-phenylalanine (4 ppm, stored at 4 °C) was added. The resulting supernatant was filtered through a 0.22 μm membrane, and the filtrate was placed in a detection vial for subsequent LC–MS analysis.

Chromatographic separation was performed on a Thermo Vanquish ultrahigh-performance liquid chromatography (UHPLC) system (Thermo Fisher Scientific, Waltham, MA, USA) using an ACQUITY UPLC® HSS T3 column (2.1 × 150 mm, 1.8 µm; Waters, Milford, MA, USA). Chromatographic conditions included a flow rate of 0.25 mL/min, column temperature of 40 °C, and an injection volume of 2 µL. In positive ion mode, the mobile phases were 0.1% formic acid in acetonitrile (C) and 0.1% formic acid in water (D), with a gradient elution program as follows: 0–1 min, 2% C; 1–9 min, 2%−50% C; 9–12 min, 50%−98% C; 12–13.5 min, 98% C; 13.5–14 min, 98%−2% C; 14–20 min, 2% C. In negative ion mode, the mobile phases were acetonitrile (A) and 5 mM ammonium formate in water (B), with a gradient elution program as follows: 0–1 min, 2% A; 1–9 min, 2%−50% A; 9–12 min, 50%−98% A; 12–13.5 min, 98% A; 13.5–14 min, 98%−2% A; 14–17 min, 2% A.

The Thermo Orbitrap Exploris 120 mass spectrometer (Thermo Fisher Scientific, USA), equipped with an electrospray ionization source, was used to acquire data in both positive and negative ion modes. The positive ion spray voltage was set to 3.50 kV, and the negative ion spray voltage to −2.50 kV, with a sheath gas of 30 arb and an auxiliary gas of 10 arb. The capillary temperature was 325 °C, with a resolution of 60,000 for full MS1 scans, scanning m/z range from 100 to 1000, and HCD was used for MS2 fragmentation with a collision energy of 30%, a resolution of 15,000, and the top 4 ions were selected for fragmentation, with dynamic exclusion to remove unnecessary MS/MS information. Mass spectrometric analyses were performed using a Thermo Orbitrap Exploris 120 instrument (Thermo Fisher Scientific) equipped with an electrospray ionization source. Data acquisition alternated between positive and negative ion modes, with spray voltages set to + 3.50 kV and −2.50 kV, respectively. The ion transfer tube temperature was maintained at 325 °C, with sheath gas and auxiliary gas flow rates set to 30 and 10 arbitrary units, respectively. Full-scan MS1 spectra (m/z 100–1000) were acquired at a resolution of 60,000, while MS2 fragmentation employed higher-energy collisional dissociation (HCD) with a normalized collision energy of 30%. Product ion spectra (MS2) were collected at 15,000 resolution using a data-dependent acquisition strategy targeting the four most intense precursor ions, with dynamic exclusion enabled to minimize redundant fragmentation.

### Metabolomics data processing and analysis

The raw mass spectrometry data files were converted to mzXML format utilizing the MSConvert tool in the Proteowizard software suite (version 3.0.8789). Subsequent data processing including peak detection, peak filtering, and peak alignment was performed using the XCMS package in the R environment, yielding quantitative compound profiles with specified parameters: bandwidth (bw) = 2, mass accuracy (ppm) = 15, peakwidth range = c (5–30 s), mass window (mzwid) = 0.015, mass difference (mzdiff) = 0.01, employing the centWave algorithm. Compound annotation was executed through cross-referencing with established public repositories (HMDB, MassBank, LipidMaps, mzCloud, KEGG) supplemented by the proprietary compound database of Suzhou Panomix Biomedical Technology Co., Ltd., maintaining a mass accuracy threshold below 30 parts per million (ppm).

Multivariate pattern recognition pipelines comprising PCA and PLS-DA were implemented via the Ropls package to compress sample data feature space. Diagnostic visualization outputs including score-loading diagram pairs and S-plot matrices elucidated inter-sample metabolite profile variations, while permutation-based cross-validation protocols monitored model overfitting risks.

Statistical validation entailed applying Wilcoxon rank-sum tests for significance probability determination, complemented by orthogonal component regression algorithms to quantify projection-axis variable importance indices (VIP) for biomarker prioritization. Metabolite classification thresholds were defined as biologically relevant at *p* < 0.05 with concurrent VIP > 1.0. Computational workflows integrated the MetaboAnalyst analytical suite with MetPA pathway repositories to detect dysregulated metabolic networks. Pathway statistical evaluations incorporated hypergeometric distributions while topological characterization utilized betweenness centrality measures. Derived through algorithmic integration of metabolite response parameters and dimensionality reduction techniques, scaled pathway activation indices enabled Pearson correlation coefficient calculations across metabolic networks. Pathway enrichment patterns were rendered via KEGG Mapper's visualization framework for differential metabolite-network mapping.

#### Multi-omics integrated analysis

Correlation analysis and heatmap visualization were performed to explore associations between microbial community composition (16S rDNA relative abundance data), metabolite profiles (metabolite relative concentrations), and aggressive behavior. Analyses utilized cloud-based platforms: OmicStudio (https://www.omicstudio.cn/) and Biodeep (https://www.biodeep.cn/). Spearman’s rank correlation coefficient was calculated to quantify statistical relationships between variables, with results filtered for significance.

#### Data analysis

Experimental measurements adopted the arithmetic mean ± SEM (standard error of the mean) formatting convention. Statistical computation environments included IBM SPSS Statistics 26.0 for hypothesis testing and GraphPad Prism 8.0 for graphical data representation. Between-group comparisons executed independent samples t-testing protocols, with probability thresholds defined through hierarchical significance stratification: *p*-values below 0.05 designating statistical significance and values under 0.01 specifying heightened confidence levels.

## Supplementary Information

Supplementary Material 1.

## Data Availability

All data generated or analyzed during this study are included in this published article and its supplementary information files.

## Abbreviations

5-HT: 5-Hydroxytryptamine
BBB: Blood-brain barrier
CNS: Central nervous system
EC: Enterochromaffin cells
FP: Feather pecking
HPA: Hypothalamic-Pituitary-Adrenal Axis
LEfSe: Linear discriminant analysis effect size
Lys: Lysine
OUT: Operational taxonomic unit
PCA: Principal component analysis
PLS-DA: Partial least squares-discriminant analysis
SCFAs: Short-chain fatty acids
TRP: Tryptophan
VIP: Variable importance
