# Supplementary material for: Integrative analysis of gut microbiota and plasma metabolites reveals mechanisms underlying aggressive behavior in chronically stressed broiler chickens
Source: Stress Biol. 2026 Apr 20;6(1):30. doi: 10.1007/s44154-026-00297-2 (PMC13096309; doi:10.1007/s44154-026-00297-2)
Supplement: Supplementary file 1 — Supplementary Material 1. [file 44154_2026_297_MOESM1_ESM.docx]

**Table S1**

Assessment of aggressive behavior in broilers

| Aggressive behavior | definition |
| --- | --- |
| Peck | The attacker forcefully pecks at the head or neck of another individual |
| Grab | The attacker uses its claws to scratch the nape and body of another individual |
| Twist | The attacker twists the neck and body of another individual with its claws |

**Table S2**

Effect of chronic corticosterone exposure on plasma and brain tissue 5-HT and DA content in broiler chickens

| Parameters | CON | CORT | P-Value |
| --- | --- | --- | --- |
| plasma |  |  |  |
| DA (pg/mL) | 11.56±2.24 | 11.55±1.56 | 0.997 |
| 5-HT (pg/mL) | 22.78±4.41 | 43.34±7.37 | 0.031* |
| Hippocampus |  |  |  |
| DA (ng/gprotein) | 217.62±22.86 | 196.27±32.52 | 0.601 |
| 5-HT (ng/gprotein) | 596.29±51.84 | 471.88±67.26 | 0.165 |
| Hypothalamus |  |  |  |
| DA (ng/gprotein) | 240±12.18 | 263.22±32.96 | 0.551 |
| 5-HT (ng/gprotein) | 1117.23±44.37 | 693.80±36.89 | 0.001** |

Note: CON: Control group, CORT: Corticosterone group; Values were expressed as means ± SEM, n = 10, **P*< 0.05, ***P*< 0.01.

**Table S3**

Pathways enriched by differential metabolites in plasma

| Pathway_name | Hits | *P*-value | Impact |
| --- | --- | --- | --- |
| Pantothenate and CoA biosynthesis | 4 | 0.0001 | 0.1333 |
| beta-Alanine metabolism | 4 | 0.0001 | 0.1250 |
| Biosynthesis of phenylpropanoids | 6 | 0.0002 | 0.0583 |
| ABC transporters | 6 | 0.0010 | 0.0438 |
| Phosphotransferase system (PTS) | 4 | 0.0014 | 0.0702 |
| Histidine metabolism | 3 | 0.0074 | 0.0638 |
| Lysine degradation | 3 | 0.0088 | 0.0600 |
| Metabolic pathways | 33 | 0.0109 | 0.0117 |
| Insulin resistance | 2 | 0.0112 | 0.1053 |
| Taurine and hypotaurine metabolism | 2 | 0.0149 | 0.0909 |
| Isoflavonoid biosynthesis | 3 | 0.0171 | 0.0469 |
| Biosynthesis of plant hormones | 3 | 0.0201 | 0.0441 |
| Carbohydrate digestion and absorption | 2 | 0.0220 | 0.0741 |
| Biosynthesis of amino acids | 4 | 0.0234 | 0.0312 |
| Arginine and proline metabolism | 3 | 0.0288 | 0.0385 |
| Taste transduction | 2 | 0.0303 | 0.0625 |
| Tryptophan metabolism | 3 | 0.0337 | 0.0361 |
| Phenylalanine, tyrosine and tryptophan biosynthesis | 2 | 0.0339 | 0.0588 |
| Lysine biosynthesis | 2 | 0.0358 | 0.0571 |
| Starch and sucrose metabolism | 2 | 0.0396 | 0.0541 |
| Central carbon metabolism in cancer | 2 | 0.0396 | 0.0541 |
| Glutathione metabolism | 2 | 0.0416 | 0.0526 |
| Vitamin digestion and absorption | 2 | 0.0436 | 0.0513 |
| Valine, leucine and isoleucine degradation | 2 | 0.0499 | 0.0476 |
| Butanoate metabolism | 2 | 0.0499 | 0.0476 |

Note: Hits indicates the number of differential metabolites enriched in the target pathway; *P*-value represents a more significant effect of the detected differential metabolites on the pathway; Impact indicates pathway impact value, larger means the more significant effect of the detected differential metabolites on the pathway,
